# Enrichment analyses of diseases and pathways associated with precocious puberty using PrecocityDB

**DOI:** 10.1038/s41598-021-83446-z

**Published:** 2021-02-18

**Authors:** Mridula Sharma, Indra Kundu, Ram Shankar Barai, Sameeksha Bhaye, Karishma Desai, Khushal Pokar, Susan Idicula-Thomas

**Affiliations:** Biomedical Informatics Center, Indian Council of Medical Research-National Institute for Research in Reproductive Health, Mumbai, 400012 India

**Keywords:** Computational biology and bioinformatics, Databases

## Abstract

Precocious puberty (PP) is an important endocrine disorder affecting children globally. Several genes, SNPs and comorbidities are reported to be associated with PP; however, this data is scattered across scientific literature and has not been systematically collated and analysed. In this study, we present PrecocityDB as the first manually curated online database on genes and their ontology terms, SNPs, and pathways associated with PP. A tool for visualizing SNP coordinates and allelic variation on each chromosome, for genes associated with PP is also incorporated in PrecocityDB. Pathway enrichment analysis of PP-associated genes revealed that endocrine and cancer-related pathways are highly enriched. Disease enrichment analysis indicated that individuals with PP seem to be highly likely to suffer from reproductive and metabolic disorders such as PCOS, hypogonadism, and insulin resistance. PrecocityDB is a useful resource for identification of comorbid conditions and disease risks due to shared genes in PP. PrecocityDB is freely accessible at http://www.precocity.bicnirrh.res.in. The database source code and content can be downloaded through GitHub (https://github.com/bic-nirrh/precocity).

## Introduction

Disease-centric databases significantly contribute to advancement of health research as they offer curated and well-annotated data amenable for further analysis. While many of the diseases such as cancers, autism, Alzheimers, PCOS and endometriosis have dedicated databases, it would be worthwhile to include more diseases in this list. In the present study, we have reviewed scientific literature on precocious puberty (PP) and developed a manually curated database with information on genes associated with PP, their ontology terms, SNPs and pathways.

Puberty is characterized by growth and maturation of primary sexual organs leading to development of secondary sexual characteristics. Puberty attained before age of 8 years in girls and 9 years in boys is considered as precocious^1^. PP is statistically defined as early pubertal development at an age that is 2.5 standard deviations lower than the mean age of pubertal onset^2^.

The onset of puberty is initiated by the maturation of hypothalamic–pituitary–gonadal (HPG) axis that ultimately leads to sexual dimorphism and fertility^3,4^. Activation of the HPG axis, nearing the pubertal age, stimulates the anterior pituitary to secrete gonadotropins such as FSH and LH, in response to which gonads develop. This process is termed as gonadarche. Gonadarche is accompanied by increased secretion of gonadal sex steroid hormones such as estrogen and testosterone. Prior to and independent of gonadarche, adrenarche occurs when there is a surge in the secretion of adrenal androgens such as DHEA and DHEA-S^5^ leading to growth and appearance of sexual hair^6–8^.

PP can be sub-classified as (i) gonadotropin dependent or central precocious puberty (CPP); (ii) gonadotropin independent or peripheral precocious puberty (PPP); and (iii) incomplete precocious puberty (IPP). CPP is caused due to premature activation of HPG axis whereas PPP is associated with increased gonadal secretion of steroidal sex hormones^9^. The overall prevalence of PP is in the range of 1: 5000 to 1: 10,000 children^7,10^. CPP is more common in girls with a female to male ratio that ranges from 3:1 to 23:1. Some rare cases of CPP triggered due to organic causes are more common among boys^11,12^. IPP comprises of variants of pubertal development like premature adrenarche, premature thelarche, and premature menarche that may lead to PP^7^.

PP is diagnosed based on physical and biochemical changes that are associated with puberty. Assessment is done using detailed family history, bone X-ray, brain MRI, and hormonal profiling^7^.

The incidence of PP is higher in individuals with central nervous system (CNS) disorders or CNS lesions^10^. Early onset of puberty profoundly impacts the psychosocial well-being of individuals^13,14^. Children with early puberty experience higher levels of behavioural and psychological disturbances as compared to children with normal puberty. Individuals with PP suffer from short stature due to premature fusion of the growth plates^15^. PP is often present with other morbidities such as McCune-Albright syndrome, congenital adrenal hyperplasia, neurofibromatosis type 1, Sturge–Weber syndrome, adrenal and ovarian tumors^12^. Premature puberty has been found to be associated with cervical, ovarian and thyroid cancers^16–18^. Few studies have reported that early menarche increases the risk of breast cancer in girls^19,20^.

The cause of PP can be ascribed to genetic, metabolic, or environmental factors. 70–80% of the variance in pubertal timing can be attributed to genetic factors^21^. Genetic studies suggest that PP has an autosomal dominant mode of inheritance^22^. Mutations in genes that are involved in sexual development such as *MKRN3*, *KISS1*, *GPR54*, *CYP19A1*, and *LHCGR* contribute to PP. Apart from genetic factors, intrauterine growth retardation and low birth weight are linked with early menarche^23^. It is unclear if childhood obesity is cause or effect of PP^23^. Exposure to endocrine-disrupting chemicals such as 1,1,1-trichloro-2,2-bis(4-chlorophenyl)ethane (DDT), monobutyl phthalate (MBP), n-nonyl phenol (n-NP), t-octylphenol (t-OP), and isoflavones like equol, genistein, and daidzein can influence hormonal dysregulation leading to PP^23,24^.

Several research groups have published valuable information on the causal factors and comorbid conditions associated with PP; this data is worthwhile to collate and analyse systematically to gain further insights on PP.

## Results

### Database content

PrecocityDB has an interactive, user-friendly web interface with options to search, browse, and visualize data. The database has curated information of 44 genes and associated 26,874 ontology terms, 235 pathways, and 199 SNPs associated with PP.

This database can be easily explored using navigation tabs on the top panel. A brief description of these tabs is given below.

1. Home: Short description of PP and information available in PrecocityDB is provided.
2. Search:
  - Quick search: Data available in PrecocityDB for a keyword can be retrieved.
  - Advanced search: Specific queries, through keywords, can be built to retrieve precise information.
3. Browse: Datasets on genes, SNPs, pathways and ontology terms can be screened.
4. SNP visualizer: Allelic variants and SNP coordinates on each chromosome can be visualized. Chromosomes 1 and 6 are associated with the maximum number of SNPs that are associated with PP (Fig. 1).

**Figure 1:**
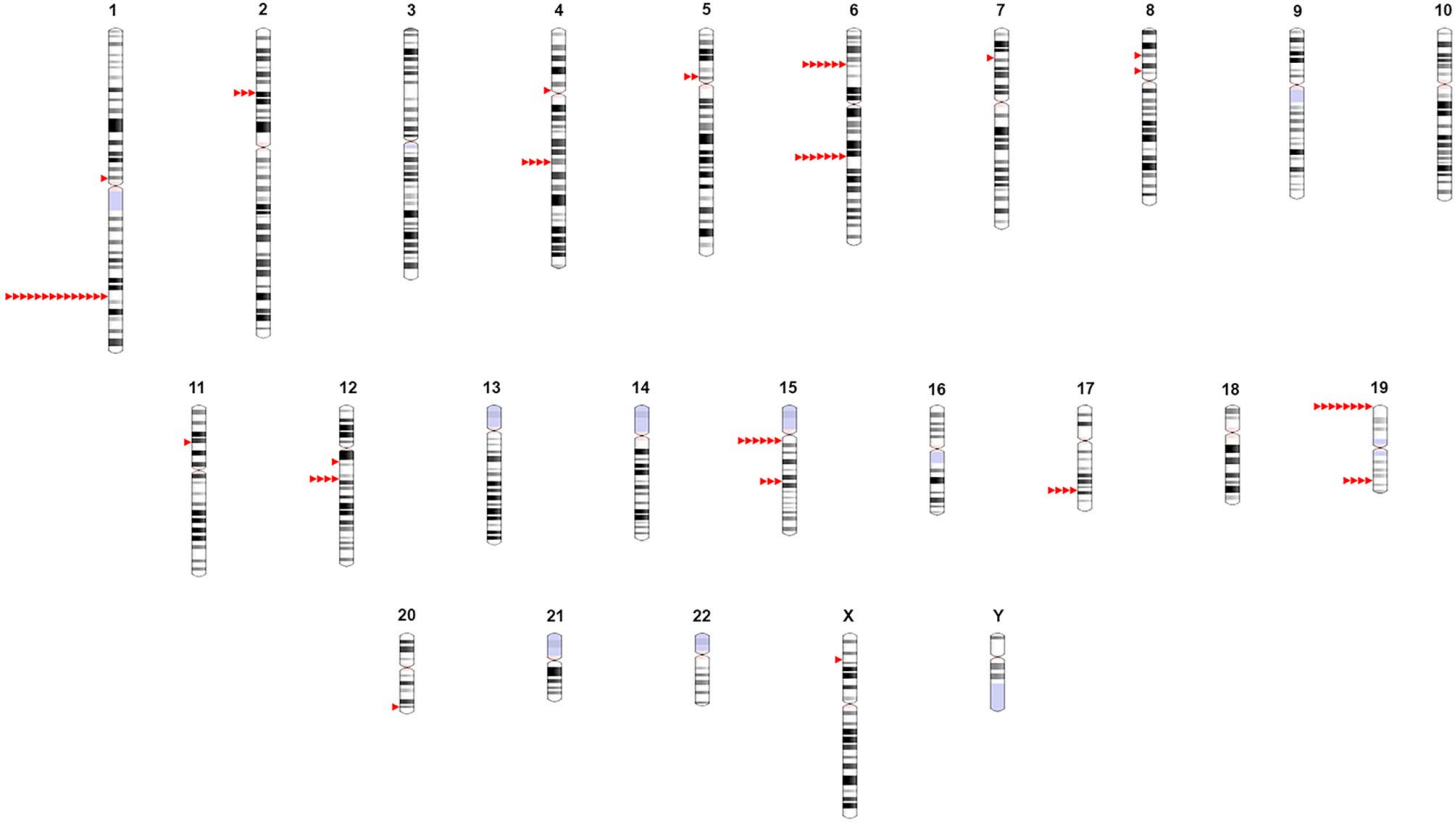
Ideogram of SNPs associated with PP. SNPs are represented by red triangles.
5. Enrichment analysis: Graphical representations of outcomes of pathway and disease enrichment analyses are provided.
6. Help: Detailed explanation of all the features in PrecocityDB and its usage, with examples, is provided.

### Data analysis

**Figure 2:**
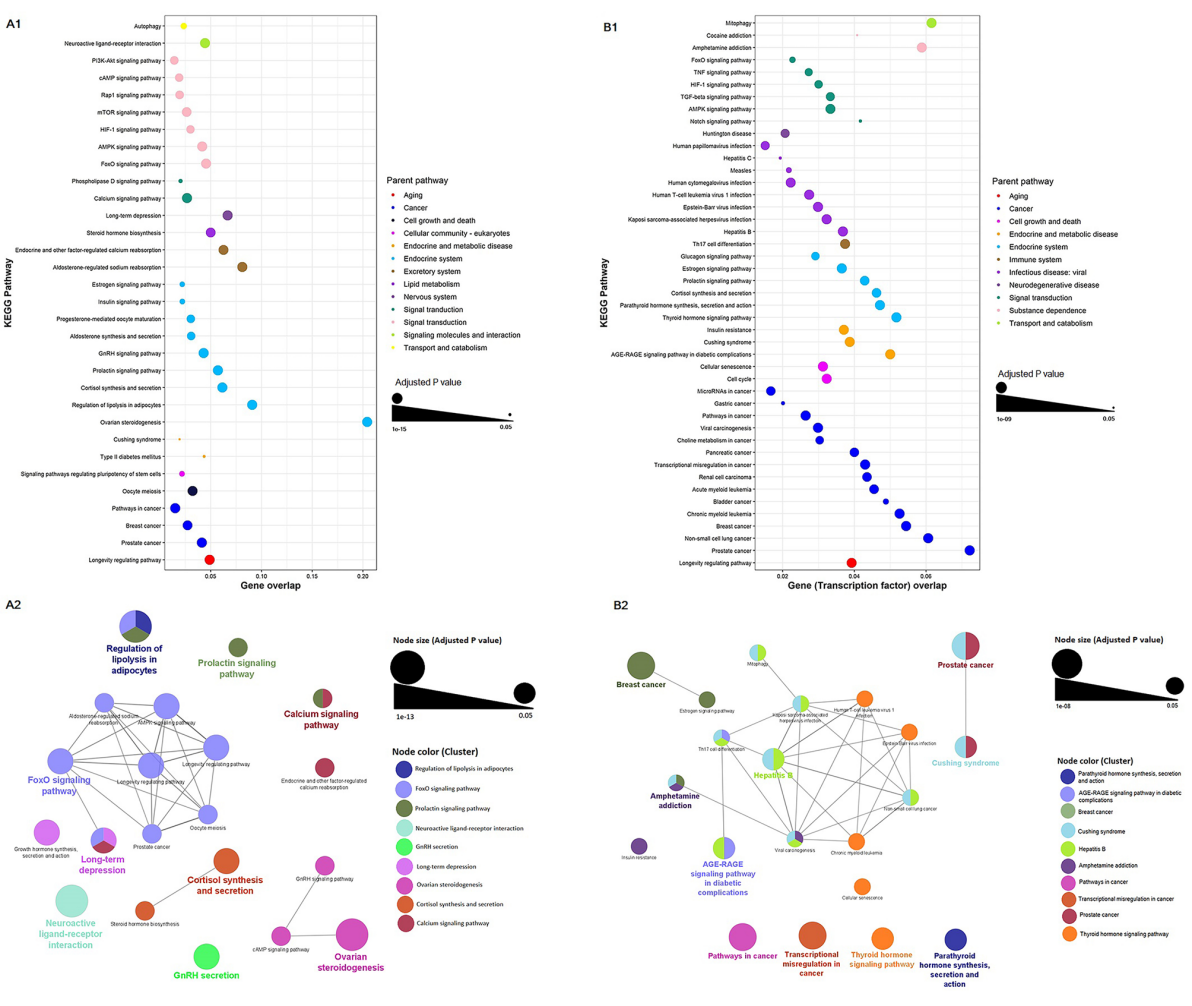
Pathway enrichment analysis using PrecocityDB gene-set (**A1**,** A2**) and transcription factors of PrecocityDB gene-set (**B1**,** B2**). In the bubble plot (generated using ggplot2 R package^25^), the x-axis represents gene overlap score (provided by Enrichr) and y-axis indicates enriched KEGG pathway terms. Bubble size is proportional to adjusted *P* value of enriched pathways. Bubble color represents parent pathway term. In the pathway networks (generated using ClueGO v2.5.7), the node size is proportional to adjusted *P* value. Edge represents at least one gene shared between two connecting nodes. Nodes within a cluster are colored based on the most significant pathway term (labelled in same color).

**Figure 3:**
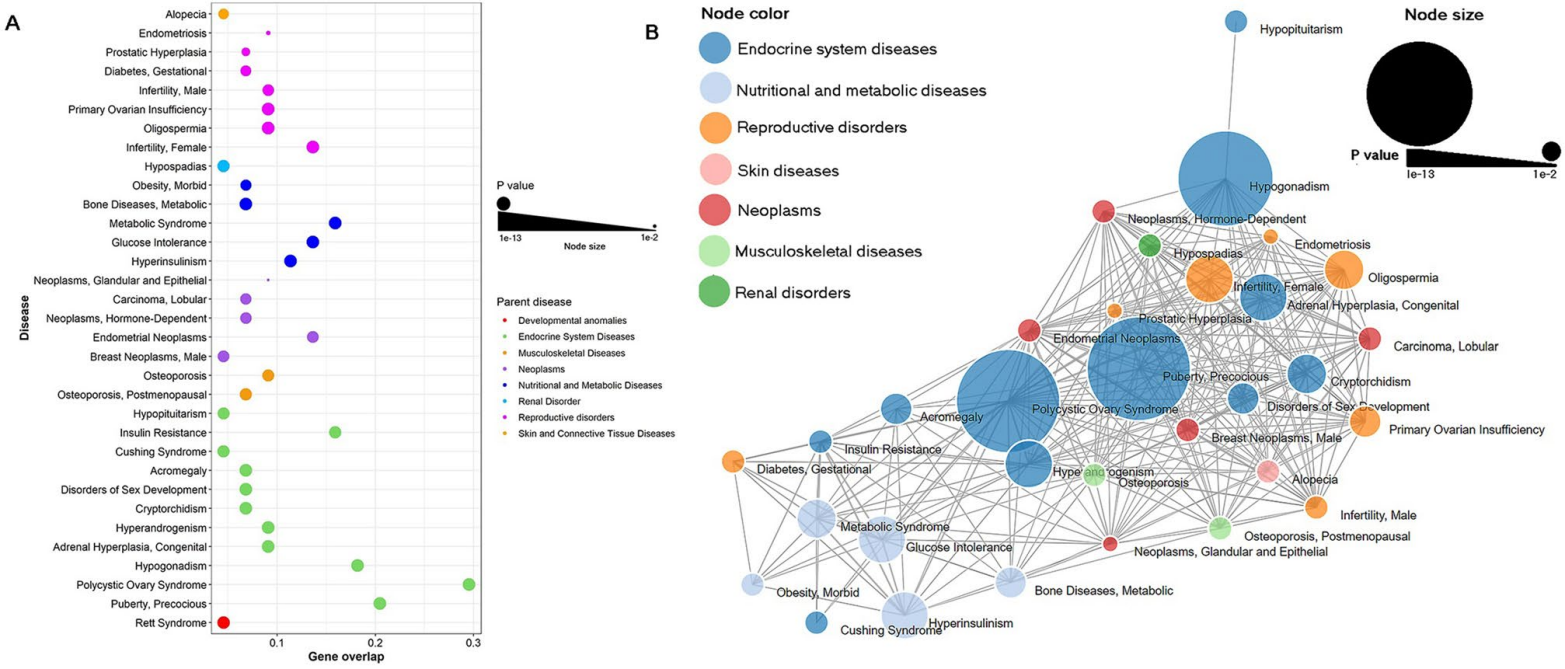
Disease enrichment analysis using enriched diseases obtained using PrecocityDB gene-set and GS2D. In the bubble plot (generated using ggplot2 R package), the x-axis represents gene overlap score (as given by GS2D) and y-axis indicates enriched disease terms. Bubble size is proportional to *P* value of enriched diseases. Bubble color represents parent disease term. In the disease-disease association network (generated using D3.js javascript library^26^), edges represent at least one gene shared between two connecting nodes. Node color represents parent disease term and node size is proportional to *P* value of disease enrichment analysis.

Pathway enrichment analysis of genes in PrecocityDB identified 32 statistically significant pathways (Fig. 2A1, Supplementary File S1, Supplementary Fig. S1A). Pathways related to endocrine system, signal transduction, and cancers were amongst the highly enriched (Fig. 2A1). Clustering analysis of these enriched pathways resulted in nine pathway clusters, of which three were interconnected and six were independent pathway clusters (Fig. 2A2). The most significant pathway clusters comprised of ovarian steroidogenesis and FoxO signalling pathway. The most significant independent pathway clusters were neuroactive ligand-receptor interactions, regulation of lipolysis in adipocytes and long-term depression. Pathway enrichment analysis of transcription factors of these genes led to identification of 45 statistically enriched pathways (Supplementary File S1, Supplementary Fig. S1B). Pathways related to viral infectious diseases, cancers, and endocrine systems were highly enriched (Fig. 2B1). Clustering analysis of the enriched pathways captured nine pathway clusters. Pathways related to cancers formed the largest clusters followed by parathyroid synthesis and thyroid hormone signalling pathways (Fig. 2B2). Disease enrichment analysis revealed that reproductive, endocrine, metabolic and cancer-related disorders were enriched in PP (Fig. 3, Supplementary File S1, Supplementary Fig. S2).

## Discussion

PP is a manifestation of hormonal dysregulation; specifically, growth hormone, gonadotropins and steroidal sex hormones^27^. These hormones are majorly responsible for endocrine and reproductive disorders such as PCOS, endometriosis, hypogonadism and infertility. These hormones are also known to influence cancer progression^28,29^. Hence, it is highly likely that individuals with PP have higher risk of developing these disorders in their life span.

In order to systematically assess the risk of co-occurrence of diseases, it is important to have a database of manually curated genes associated with PP. Since databases on PP were not available in the public domain, we developed PrecocityDB for providing researchers a high quality database on information related to PP that is curated from scientific publications.

Through the curation process, we identified 44 genes and 199 SNPs associated with PP. The highest number of SNPs associated with PP was reported for *MKRN3* (Makorin ring finger protein 3) followed by *LHCGR* (luteinizing hormone (LH)/choriogonadotropin receptor gene)*, KISS1* (Kisspeptin)*, KISS1R* (Kisspeptin receptor)*, and LIN28B* (Lin-28 Homolog B)*. MKRN3* gene is the most widely studied gene for its association with precocious puberty^30^. It has ubiqutin protein ligase activity. Mutation in *MKRN3* inhibits proteolysis of proteins involved in activation of GnRH neurons^31^. Mutations in *MKRN3*, *KISS1* and *KISS1R* contribute to precocious puberty by activating gonadotropic axis^32^. Inactivating *LHCGR* mutations in females lead to amenorrhea and infertility, whereas activating *LHCGR* mutations in males have been associated with familial male-limited precocious puberty (FMPP)^33^. *LIN28B* expression influences the timing of major developmental events and is thus associated with pubertal onset. Loss of function mutations in *LIN28B* gene are known to contribute to precocious development^34,35^.

To evaluate the comorbidity risk, disease and pathway enrichment analyses of gene-set in PP were performed. Some of the top pathways identified were ovarian steroidogenesis, GnRH signalling, estrogen signalling, and thyroid hormone signalling pathways (Fig. 2); these pathways are known to be critical for PP, PCOS and cancers^36–40^. In order to further validate these observations, disease enrichment analysis was performed based on significant co-occurrences of PP gene-set and diseases using GS2D tool^57^. It is noteworthy that reproductive, endocrine, metabolic and cancer-related disorders were enriched (Fig. 3).

The above findings are in good agreement with clinical observations of patients with PP. Franceschi et al. investigated the prevalence of PCOS in a cohort of young women with previous idiopathic central precocious puberty (ICPP) and observed that patients with ICCP were prone to develop PCOS in their adulthood^40^. Bodicoat et al., through their large cohort study, had investigated the association of pubertal age and risk of developing breast cancer. An increased risk of breast cancer was associated with incidences of earlier thelarche, menarche, early regular periods, a longer time between thelarche and menarche, or a shorter time between menarche and the onset of regular periods^41^. Bonilla et al., using a Mendelian randomization approach, observed that attainment of sexual maturity at a younger age than normal poses a significant threat of developing prostate cancer in adolescent boys^42^.

Enrichment analysis performed using the genes present in PrecocityDB helped to identify the well-studied pathways of PP (Fig. 2). Additionally, enrichment analysis could also identify genes and pathways that are not yet reported to be associated with PP based on human studies. For example, although there is evidence suggesting the role of *FOXO3* in early pubertal development in rodents^43–45^, *FOXO* gene family is not included in PrecocityDB as its association with PP based on human studies is not yet reported. Interestingly, FoxO signalling pathway was found to be one of the enriched pathways based on enrichment analysis of PrecocityDB genes because of the overlapping genes from related pathways such as insulin signalling pathway, MAPK signalling and PI3K-Akt signalling pathway.

PrecocityDB will be updated with time and as more genes get validated for its association with PP; this will lead to more accurate results for enrichment analysis. However, even with the present data the enrichment analysis does give out interesting insights and testable hypothesis for the scientific community.

## Conclusion

PP adversely affects the quality of life of adolescents and could have long-term health consequences. In order to comprehend the comorbidity risk in PP due to shared genes and biochemical pathways, it is essential to have a high quality database of genes and SNPs that are associated with PP. PrecocityDB is the first online database that catalogues genetic and polymorphism data associated with PP along with relevant reference literature. Pathway enrichment analysis using the PrecocityDB gene-set indicated that individuals with PP are at higher risk of developing reproductive and metabolic disorders such as PCOS, hypogonadism, insulin resistance, metabolic syndrome, glucose intolerance and hyperinsulinism. Pathways related to prostate cancer, breast, endometrial and hormone-dependent neoplasms were also found to be enriched in the PP gene-set. The hypotheses generated based on gene enrichment analysis are consistent with existing clinical observations. The clinical data on longitudinal prognosis for individuals with PP and diseases that are more likely to emerge in adulthood is scarce. This research gap needs to be addressed for evidence-based management of PP.

## Methodology

### Creation of gene dataset

**Figure 4:**
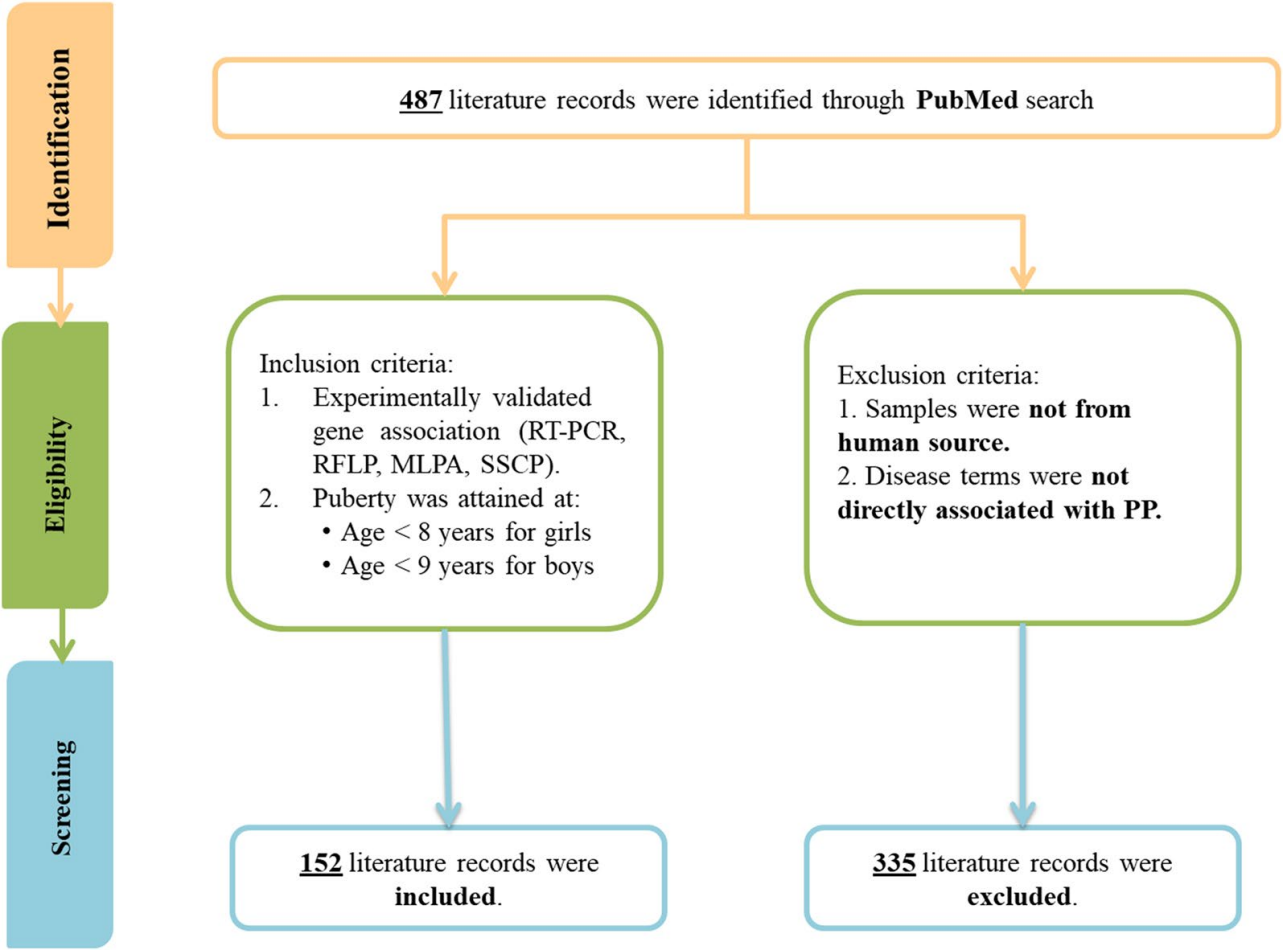
Schema for creation of gene dataset.

PubMed was searched on 3rd July 2020 using MeSH (Medical Subject Headings) terms associated with PP such as, "Precocious Puberties", “Premature Pubarche” , "Puberties, Precocious", "Pubertas Praecox", "Praecox, Pubertas", "Precocious Puberty", "Precocious Puberty, Central", "Central Precocious Puberties", "Central Precocious Puberty", "Precocious Puberties, Central", "Puberties, Central Precocious", "Puberty, Central Precocious", "Sexual Precocity", "Precocities, Sexual", "Precocity, Sexual", "Sexual Precocities", "Idiopathic Sexual Precocity", "Idiopathic Sexual Precocities", "Precocities, Idiopathic Sexual", "Precocity, Idiopathic Sexual", "Sexual Precocities, Idiopathic", "Sexual Precocity, Idiopathic", "Familial Precocious Puberty", "Familial Precocious Puberties", "Precocious Puberties, Familial", "Precocious Puberty, Familial", "Puberties, Familial Precocious", "Puberty, Familial Precocious", "Precocious Puberty, Male-Limited", "Male-Limited Precocious Puberties", "Male-Limited Precocious Puberty", "Precocious Puberties, Male-Limited", "Puberties, Male-Limited Precocious", "Puberty, Male-Limited Precocious", "Precocious Puberty, Male Limited", "Testotoxicosis" and "gene". Using this query, 487 literature records were retrieved from PubMed. These publications were reviewed to confirm gene association with PP (Fig. 4).

The studies were included in PrecocityDB if (a) gene association with PP was confirmed based on experiments such as Real Time-Polymerase Chain Reaction(RT-PCR), Restriction fragment length polymorphism (RFLP), Multiplex ligation-dependent probe amplification (MLPA), Single-Strand Conformation Polymorphism(SSCP) and (b) puberty was attained at age less than 8 years for girls and less than 9 years for boys. Studies were excluded if (a) samples were not from human source; and (b) disease terms were not directly associated with PP; such as “pseudo-precocious puberty”, “premature/precocious adrenarche”, “premature/precocious thelarche”, “congenital adrenal hyperplasia”, “McCune Albright Syndrome”, “21-hydroxylase deficiency”, “androgen excess syndrome”, “aromatase excess syndrome”, “hyperandrogenism”, and “hirsutism” (Fig. 4).

Relevant data, such as nature of study population, ethnicity, hereditary information, and mutations/SNPs were appended to gene records based on evidence available in literature. The gene records were annotated for additional information such as unique identifiers for gene and protein, protein structures, family and ontology term details, metabolic pathway information by mapping them to databases such as NCBI^46^, dbSNP^47^, Ensembl^48^, UniProt^49^, GO^50^, KEGG^51^, PDB^52^ and, Reactome^53^.

### Pathway enrichment analysis

Pathway enrichment analysis was performed on the manually curated gene-set and its transcription factors using Enrichr^54^, a gene list enrichment analysis tool which is independent of expression data and based on Fisher’s exact test. The transcription factors of the gene-set were identified using TRRUST V2^55^. KEGG database was used as pathway resource. Pathways with adjusted *P* value < 0.05 were selected. Enriched pathways were mapped to parent pathway groups using KEGG. The pathways were clustered based on KEGG pathway terms using ClueGO v2.5.7 with a minimum of two genes per term^56^.

### Disease enrichment analysis

Gene-disease associations were derived for PP gene-set based on statistically significant co-occurrences of genes and diseases in PubMed by using the GS2D tool^57^. Diseases with *P* value and FDR < 0.05 were selected. Enriched diseases were mapped to parent disease terms using a rule-based method adapted from PCOSKB_R2_^58^.

### Database interface development

PrecocityDB was developed using PHP 7.2.24, MariaDB Server 10.1.44, JavaScript, ideogram.js v1.22.0, and XHTML 1.0 and hosted on Apache webserver.

### SNP visualizer

This tool can be used for visualization of chromosomal coordinates of SNPs associated with PP. Ideogram.js JavaScript library^59^ was used for creation of SNP visualizer. Chromosomes, in haploid state, are shown as vertical bars. Black and grey bands represent heterochromatin region; lighter bands represent euchromatin region; and blue represents variable region. Centromere is marked in pink. Users can select a shape from list of shapes (circles, triangles or rectangles) to display SNPs associated with PP. The gene name, SNP ID and chromosomal location of each SNP can be perused by hovering over the SNP pointer.

## Supplementary information

Supplementary information 1.

Supplementary information 2.
