# Supplementary material for: Enrichment analyses of diseases and pathways associated with precocious puberty using PrecocityDB
Source: Sci Rep. 2021 Feb 18;11:4203. doi: 10.1038/s41598-021-83446-z (PMC7893021; doi:10.1038/s41598-021-83446-z)
Supplement: Supplementary file 2 — Supplementary information 2. [file 41598_2021_83446_MOESM2_ESM.docx]

**Enrichment analyses of diseases and pathways associated with precocious puberty using PrecocityDB**

Mridula Sharma^1¥^, Indra Kundu^1¥^, Ram Shankar Barai^1^, Sameeksha Bhaye^1^, Karishma Desai^1^, Khushal Pokar^1^, Susan Idicula-Thomas^1^*

*To whom correspondence should be addressed:

Dr. Susan Idicula-Thomas, E-mail: [thomass@nirrh.res.in](mailto:thomass@nirrh.res.in), Tel: 91**-** 2224192107/04

^¥^The authors wish it to be known that, in their opinion, the first two authors should be regarded as Joint First Authors.

^1^Biomedical Informatics Center, Indian Council of Medical Research-National Institute for Research in Reproductive Health, Mumbai, 400012, India

**SUPPLEMENTARY DATA**

**Supplementary Figures:**


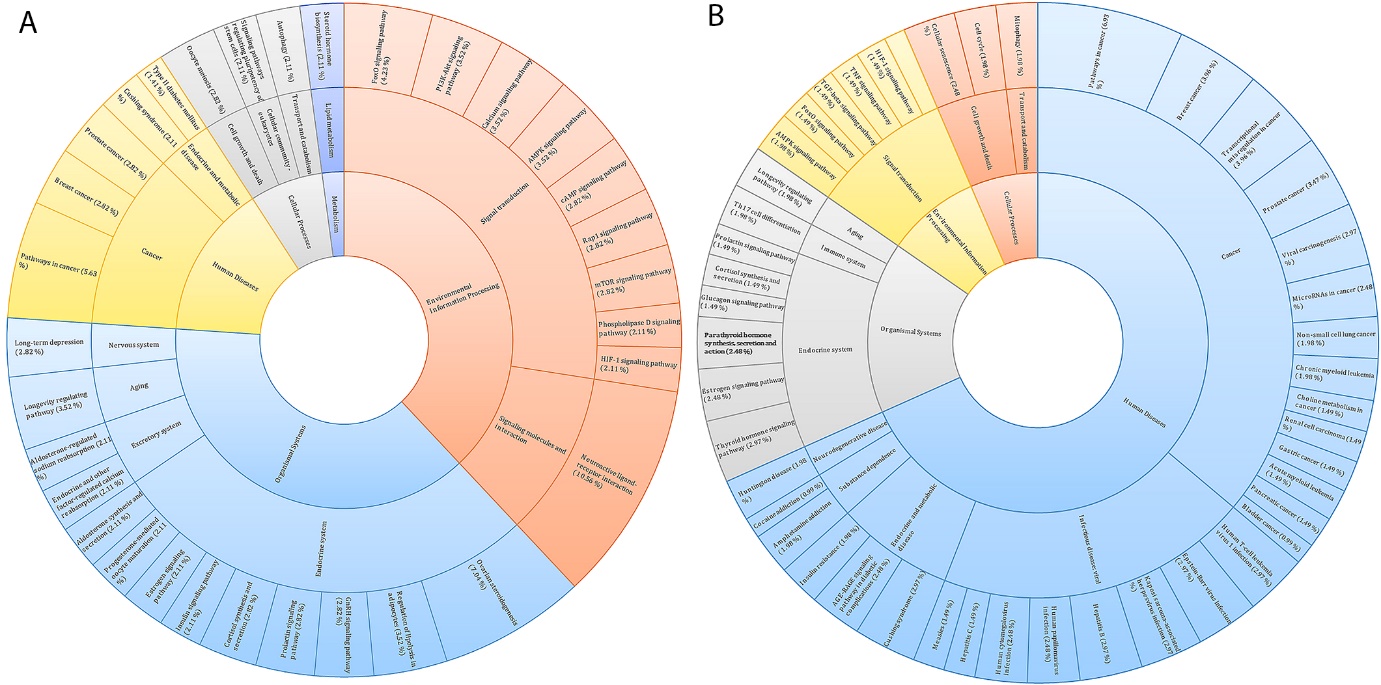


Supplementary Figure 1: Multi-level pie chart illustrating percent distribution of enriched KEGG pathways along with its parent terms obtained using (A) PrecocityDB gene-set and (B) transcription factors of PrecocityDB gene-set.


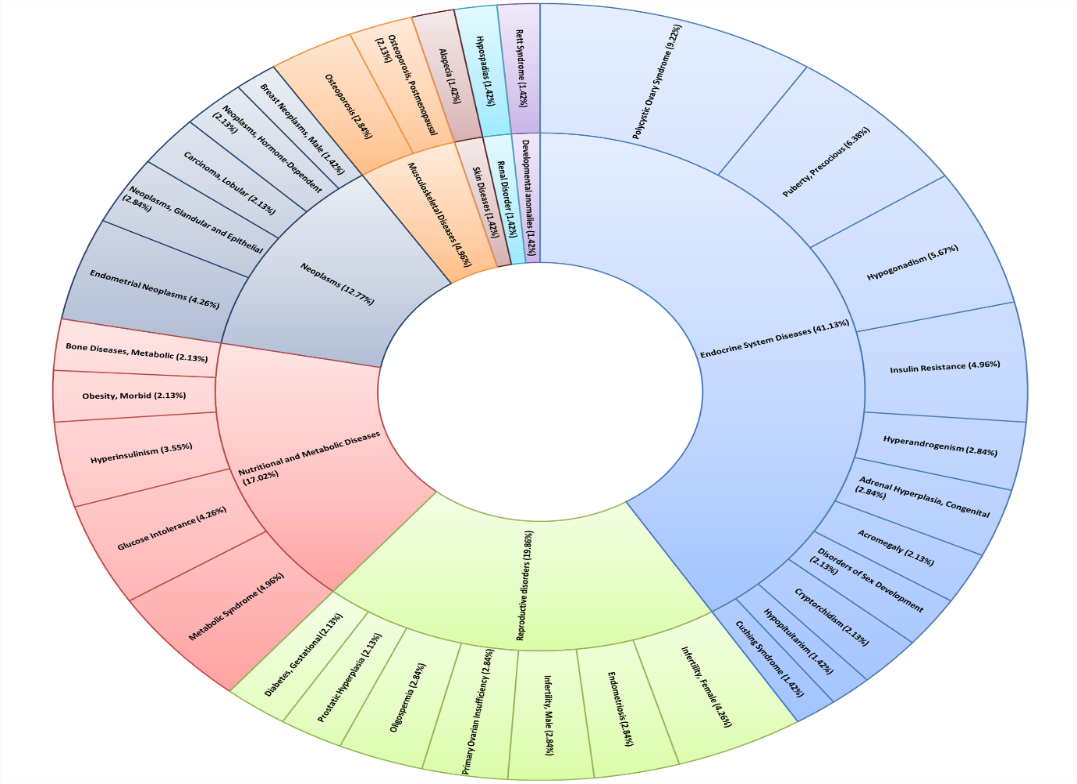


Supplementary Figure 2: Multi-level pie chart illustrating percent distribution of enriched diseases obtained using PrecocityDB gene-set and GS2D.
